# Mechanism and consequences for avoidance of superparasitism in the solitary parasitoid *Cotesia vestalis*

**DOI:** 10.1038/s41598-020-67050-1

**Published:** 2020-07-10

**Authors:** Wen-bin Chen, Liette Vasseur, Shuai-qi Zhang, Han-fang Zhang, Jun Mao, Tian-sheng Liu, Xian-yong Zhou, Xin Wang, Jing Zhang, Min-sheng You, Geoff M. Gurr

**Affiliations:** 1 State Key Laboratory of Ecological Pest Control for Fujian and Taiwan Crops, Institute of Applied Ecology, Fujian Agriculture and Forestry University, Fuzhou, China; 2 Joint International Research Laboratory of Ecological Pest Control, Ministry of Education, Fuzhou, China; 3 Department of Biological Sciences, Brock University, St. Catharines, Ontario Canada; 4 Graham Centre, Charles Sturt University, Orange, New South Wales Australia

**Keywords:** Behavioural ecology, Entomology

## Abstract

A parasitoid’s decision to reject or accept a potential host is fundamental to its fitness. Superparasitism, in which more than one egg of a given parasitoid species can deposit in a single host, is usually considered sub-optimal in systems where the host is able to support the development of only a single parasitoid. It follows that selection pressure may drive the capacity for parasitoids to recognize parasitized hosts, especially if there is a fitness cost of superparasitism. Here, we used microsatellite studies of two distinct populations of *Cotesia vestalis* to demonstrate that an egg laid into a diamondback moth (*Plutella xylostella*) larva that was parasitized by a conspecific parasitoid 10 min, 2 or 6 h previously was as likely to develop and emerge successfully as was the first-laid egg. Consistent with this, a naive parasitoid encountering its first host was equally likely to accept a healthy larva as one parasitized 10 min prior, though handling time of parasitized hosts was extended. For second and third host encounters, parasitized hosts were less readily accepted than healthy larvae. If 12 h elapsed between parasitism events, the second-laid egg was much less likely to develop. Discrimination between parasitized and healthy hosts was evident when females were allowed physical contact with hosts, and healthy hosts were rendered less acceptable by manual injection of parasitoid venom into their hemolymph. Collectively, these results show a limited capacity to discriminate parasitized from healthy larvae despite a viability cost associated with failing to avoid superparasitism.

## Introduction

Host selection and discrimination are essential behaviors of parasitoids^1^. Encountering a parasitized host may evoke different responses between gregarious and solitary parasitoid species^2^. In gregarious parasitoids, multiple eggs from one or more females can be laid in the host and complete development. In contrast, solitary parasitoids usually lay only one egg per host that reaches adulthood^3^. These parasitoids are generally predicted to avoid already parasitized (superparasitized) hosts because the earliest laid parasitoid larva may have to compete with or directly kill a subsequently laid newest competitor^4,5^.

Superparasitism is a common phenomenon in solitary parasitoids^6^. For a long time, most researchers believed that laying more than one egg in a host was sub-optimal and regarded as a mistake in the case of solitary parasitoids^6,7^. Superparasitism can, however, be adaptive under some conditions^6,7^. In parasitoid *Microplitis rufiventris* (Hymenoptera: Braconidae), superparasitism increases the success rate of emergence of wasp larvae when using a suboptimal host (advanced host)^8^. In the parasitoid *Spalangia cameroni* (Hymenoptera: Pteromalidae), when hosts are scare, superparasitism is observed and offspring production per parasitized host increases^9^. Superparasitism can help larvae of the parasitoid *Pseudapanteles dignus* evade host encapsulation^10^. Parasitoids are thought to use encounters with unparasitized hosts as a source of information to evaluate host density in the habitat^11^. Clearly, such responses can only happen when the parasitoid has the capacity to discriminate parasitized from unparasitized hosts, a phenomenon described as host discrimination^12^.

In parasitoid wasps, external or internal chemical cues are thought to play key role in host discrimination^13–16^. These cues, also referred as host marking pheromones (HMP), can be detected by conspecific’s antennae in the case of external cues^17^ or by ovipositors to detect internal cues^13,18^. External cues have been described in multiple parasitoid species^19–21^ such as *Anaphes victus* (Hymenoptera: Mymaridae), allowing other females to reject previously-parasitized hosts without antennal probing^22^. In contrast, the parasitoid *Leptopilina heterotoma* (Hymenoptera: Eucoilidae) larva employs internal cues to discriminate hosts after ovipositor insertion^23^. Even though the cues can be used for host discrimination with some variation, it is believed that hemolymph composition, may be one of the most important cues for parasitized host discrimination^24^.

*Cotesia vestalis* (Hymenoptera: Braconidae) is an internal and solitary parasitoid that can successfully parasitize 2^nd^ to 4^th^ larval instars of its main host, the globally significant brassica pest, diamondback moth, *Plutella xylostella* (Lepidoptera: Plutellidae) although it prefers 2^nd^ and 3^rd^ instars^25–27^. *Cotesia vestalis* is a typical hymenopteran parasitoid in that unfertilized eggs develop into males and fertilized eggs develop into females^28^. The eggs of *C. vestalis* hatch about 36 h to 48 h after oviposition^29,30^, and the duration of egg and larval stage is about 6.8 days^29^. The longevity of *C. vestalis* adult averages 10–15 days with food supplied, and the expected maximum parasitization rate of females is about 30 hosts per day^31^. *C. vestalis* is known to use chemical cues such as the plant volatiles to locate the host habits^32,33^, and the antenna and ovipositor play important roles in host recognition^32^.

Superparasitism is common in *C. vestalis*. When this occurs, the larvae use their mandibles to attack other parasitoid larvae present in the host and the first-hatched generally considered to have the best opportunity for success^30^. There is little evidence showing that a larva emerging from a second egg can successfully develop when superparasitism occurs in *C. vestalis*, suggesting a strong selective advantage of avoiding superparasitism. This parasitoid is used worldwide as a biological control agent of *P. xylostella*, so it is of practical as well as scientific interests to establish the significance of superparasitism. Accordingly, we investigated the effects of superparasitism and the capacity of parasitoid females to discriminate between healthy and parasitized hosts. We hypothesized that there would be a measurable fitness cost to *C. vestalis* of superparasitism and this would drive selection for at least some capacity to recognize superparasitized host larvae based on female HMPs.

## Materials and Methods

### Insects

Both *P. xylostella* and *C. vestalis* were maintained and tested in environmental chambers at 25 °C ± 2 °C, 60% ± 10% RH, and a photoperiod of L14:D10. Founders of *P. xylostella* and a Fuzhou strain (FZ strain) of *C. vestalis* cultures were sourced from pupae/cocoons collected from a cabbage field in Nantong Town, Fuzhou, China (25.95°N, 119.27°E), in May 2014. A second strain of *C. vestalis* was obtained from Zhejiang University in Hangzhou (Zhejian population, ZJ strain) (30.27°N, 120.15°E) that originated from that more northerly part of Eastern China. Both strains were reared in laboratory for more than two years; the earlier observations showed that they had similar development, parasitism rate, and body size. Both strains were used in experiment 1.1. For all other experiments only the FZ strain was used. The *P. xylostella* population was reared on radish sprouts and the *C. vestalis* population was reared on larvae of *P. xylostella*, both for more than 20 generations before being used for experiments.

### Mating and parasitism procedures

Newly emerged *C. vestalis* females were placed individually in plastic tubes (diameter: 8 cm, length: 11 cm) each containing a newly emerged male. A drop of honey and cotton ball soaked with water was placed on the top of the tube for parasitoid feeding and left for 24 h. Preliminary studies had established that after 24 h under these conditions, over 95% of females produced female progeny, indicating they had effectively mated. All the females used for parasitism and superparasitism experiments were previously mated. After 24 h, the female was transferred into a glass tube (diameter: 13 mm, length: 55 mm) with a drop of honey on the side. The *P. xylostella* larvae were individually introduced into the glass tube for parasitism. Earlier work showed that >93% of ovipositor insertions lead to oviposition by *C. vestalis* (refer to Chen *et al*.^34^).

#### Survival of superparasitized eggs

To test whether a second-laid egg could develop into an adult, a study was conducted using both the Zhejiang (ZJ) and the Fuzhou (FZ) strains. A population of 18 ZJ and another population of 22 FZ mated females were used as maternal generation. A third instar *P. xylostella* larva was introduced in a glass tube with one female *C. vestalis* ZJ-strain (selected randomly from ZJ population) for oviposition. After 10 minutes, the ZJ-strain female was removed and replaced with a female *C. vestalis* of FZ-strain (selected randomly from ZJ population) to obtain conspecific-superparasitism. The same procedure was repeated with intervals between parasitism and superparasitism of 2, 6 and 12 h (each interval time was used 40 larvae). All superparasitized larvae from the same interval treatment were reared together in a plastic chamber (with a mesh roof, diameter: 15 cm, height: 8 cm) until pupation or death. The sex of emerged *C. vestalis* offspring was identified and recorded, the offspring were then individually placed in Eppendorf tube and stored at -80 °C to be used for DNA extraction. All the mothers and their offspring from the different time intervals were genetically assessed using seven microsatellites^35^. GENALEX v6.5^36,37^ was used to determine whether the offspring belonged to the ZJ or FZ strain (see supplementary material for detail). Since only one adult can emerge from parasitism from a solitary parasitoid, it was predicted that ≦40 offspring (ZJ + FZ) would emerge. The proportions of emergence of ZJ or FZ strain offspring in the same time interval treatment were compared using Chi-Square test.

#### Effect of superparasitism on progeny production and bionomics

In order to test whether superparasitism affected progeny when the interval between parasitism and superparasitism was short, *P. xylostella* larvae were exposed to three types of parasitism (parasitism, self-superparasitism, conspecific-superparasitism), using FZ strain females. For the parasitism treatment, 3^rd^-instar *P. xylostella* larvae were individually exposed to only one parasitoid with one oviposition event allowed then reared individually until the emergence of the parasitoid.

For the self-superparasitism, a 3^rd^-instar *P. xylostella* larva was first exposed to a female parasitoid for oviposition, and then kept with the same parasitoid for a second oviposition event (interval within 10 min). The larva was then transferred into a Petri dish to be reared until emergence of the parasitoid. For the conspecific-superparasitism, a 3^rd^-instar *P. xylostella* larva was first exposed to a female parasitoid that oviposited. This parasitoid was then removed and replaced with another naive female parasitoid to oviposit (interval within 10 min). For each treatment, the procedure was repeated with 60 larvae. Once completed, larvae were transferred into a Petri dish until emergence. The duration of each host larval stage until the emergence of the parasitoid cocoon was recorded, as well as the weight of the cocoon (within 24 h of pupation). The cocoons were then left to emerge. The emerged parasitoids were reared individually in a Petri dish with 20% honey solution. After parasitoid death, the lengths of the left hind tibia and the left wing were measured under a calibrated stereomicroscope (Olympus-SZX2) with the software “cellSens dimension” (Olympus Ltd, Tokyo, Japan) to quantify body size. Life history traits of the progeny from three treatments were compared using a General Linear Model (GLM) with treatment being the main factor.

#### Discrimination between parasitized and unparasitized larvae

To obtain parasitized *P. xylostella* larvae, FZ strain *C. vestalis* mated females (20 replicates) were used for parasitism, and the larvae were used for the following two experiments (1.3.1 and 1.3.2).

Comparing rate of oviposition of *C. vestalis* on unparasitized and parasitized *P. xylostella* larvae Oviposition rate of *C. vestalis* was examined when the parasitoid was exposed to 1) larvae that were not parasitized (n = 86), 2) larvae parasitized 10 min prior to the start of the experiment (n = 67), and 3) larvae parasitized 12 h prior to the start of the experiment (n = 52). To better understand whether repeated encounters of such larvae could influence the rate of oviposition, the same female parasitoid was presented to three different larvae over a period of three minutes. In the first minute, a *P. xylostella* larva from one of the three treatments was introduced into a tube (diameter: 13 mm, length: 55 mm). Subsequent acceptance (i.e., insertion of the ovipositor) or rejection of the larva within the following 20 sec was recorded computer-based video recording system installed in an animal behavior observing chamber, as described by Chen *et al*.^34^. After 30 sec, the larva was removed. In the second minute, a new *P. xylostella* larva from the same treatment was introduced to the same parasitoid. The oviposition or rejection was again recorded, and the second larva was removed following the same procedure than in the first minute. In the third minute, a third larva (same treatment) was introduced, following the same procedure. Data were analyzed using a Generalized Linear Mixed Model (GLMM) and binary logistic regression as target distribution and relationship (link) with the linear model, with “acceptance (0)” as the reference value. In this, treatment served as main factor and the sequence was the second linked variable. Differences in acceptance rate in each encounter point were compared by Chi-square test. The Bonferroni modification was applied with the correction of *P* values and alpha level. Analyses were conducted using SPSS version 24.

Discrimination by *C. vestalis* females between self-parasitized and conspecific-parasitized *P. xylostella* larvae To determine whether *C. vestalis* females could discriminate between self-parasitized and conspecific-parasitized hosts, the following procedure was followed. Fifty-six female parasitoids were used for self-superparasitism test and 58 for conspecific-superparasitism test. Each female parasitoid was allowed to parasitize three unparasitized 3^rd^-instar *P. xylostella* larvae in a tube, after which the larvae parasitized by this specific wasp were placed in a glass tube (the tube was marked with the same number as the parasitoid) for the following treatments after a period of rest of 10 min. For the self-superparasitism treatment, the parasitoid that was previously exposed to the three larvae (tube with the same number) was again exposed to these larvae following the same procedure as described in section 1.3.1 i.e., the three larvae were sequentially exposed to the parasitoid over a period of three minutes. For conspecific-superparasitism treatment, the parasitoid was presented to three larvae that were parasitized by a different parasitoid in sequence, using the same procedure as self-superparasitism. The superparasitism decision (acceptance or rejection) was recorded as previously described. Statistical analyses were as described in 1.3.1.

#### Effect of topical application of hemolymph on parasitism acceptance by naive parasitoids

In order to test whether topical application of hemolymph influenced host acceptance by *C. vestalis*, the hemolymph from parasitized *P. xylostella* larvae was isolated and applied onto the cuticle of unparasitized larvae. Third instar *P. xylostella* larvae that had been parasitized 10–30 minutes previously were pricked with a drawn-glass needle and the hemolymph collected by pressing the larva. Then the hemolymph was immediately placed onto unparasitized *P. xylostella* larvae using an insect dissecting needle. Larvae were then exposed to *C. vestalis* within 10 min of being treated prior to any visual sign of melanisation of the hemolymph. The control consisted of the hemolymph from unparasitized larvae, collected and applied with the same method as parasitized larvae. The acceptance and rejection rates were recorded for 53 inexperienced (mated but not prior oviposition) parasitoid females when exposed to unparasitized larvae spread with unparasitized larval hemolymph, and for 51 inexperienced parasitoid females exposed to unparasitized larvae but spread with parasitized larval hemolymph. The acceptance and rejection rates were recorded using the criteria previously described. A GLMM was used to compare the difference of parasitoid’s parasitism decision between larvae applied with unparasitized larval hemolymph or with parasitized larval hemolymph.

#### Effect of topical application of hemolymph on parasitism acceptance by experienced parasitoids

To determine whether having oviposition experience may influence acceptance or rejection rates when encountering unparasitized larvae with larval hemolymph from unparasitized or parasitized larvae, a similar experiment as in section 1.4 was conducted with parasitoids that oviposited 10 minutes before. Fifty-four experienced parasitoid females were presented to unparasitized larvae covered with unparasitized larval hemolymph, and 52 experienced parasitoid females were exposed to unparasitized larvae covered with parasitized larval hemolymph. The acceptance and rejection rates were recorded using the criteria previously described. A GLMM was used to compare the difference of parasitoid’s parasitism decision between larvae applied with unparasitized larval hemolymph or with parasitized larval hemolymph. Differences in acceptance rate in each encounter point were compared by Chi-square test.

#### Influence of hemolymph injection from parasitized larvae on parasitism acceptance or rejection rate of inexperienced parasitoid

In order to test whether discrimination of the parasitoid between parasitized and unparasitized larvae was affected by non-topical hemolymph-mediated effects, unparasitized host larvae were injected with the hemolymph of either parasitized or unparasitized larvae. In this experiment, we used early stage 4^th^ instar *P. xylostella* larvae for injection and hemolymph collection. For the parasitized treatment, donor larvae were observed to have been stung three times. The hemolymph was then collected as described in the previous experiment but placed in an Eppendorf tube in an ice-bath, immediately centrifuged at 12000 g for 1 min, and the resulting upper-liquid was extracted for the treatment. The extract was injected into unparasitized larvae by a nanoliter injector (NANOLITER 2010 (World Precision Instruments Inc, Florida, USA)) at 70 nl/larva. The hemolymph from unparasitized larvae was used for injection as control treatment. The procedure of three encounters of larvae by *C. vestalis* was used to determine acceptance or rejection as described in 1.3.1. Fifty-two female parasitoids were exposed to larvae injected with parasitized host’s hemolymph, and 53 were exposed to larvae injected with unparasitized host’s hemolymph. The data of parasitism decisions of the two treatments were compared by GLMMs. Differences in acceptance rate in each encounter point were compared by Chi-square test.

#### Effect of parasitoid venom on parasitism acceptance/rejection rate of inexperienced parasitoids

As far as we know, *C. vestalis* injects venom and polydnavirus (PDV) into the host during oviposition and, because *C. vestalis* was observed to reject hosts parasitized only minutes previously, we selected venom as the likely marking component in this step of experiment. We tested whether venom was the factor for oviposition decision of a new parasitoid female, venom was collected from 10 mated *C. vestalis* females that were younger than 5 days from hatching. Parasitoids were frozen at -50 °C for 3 min then surface-sterilized with 75% ethanol and sterilized-deionized water. The parasitoids were then dissected to collect the intact venom reservoir, which was then ruptured using dissecting forceps in 20 μl ddH_2_O on a Petri dish. Venom was collected in an Eppendorf tube and centrifuged at 12000 g for 1 min, the venom reservoir tissue discarded, and the supernatant used for injection at 70 nl/larva. Early stage 4^th^ instar *P. xylostella* larvae were used for injection and be exposed to inexperience parasitoids (n = 51). For the control treatment (parasitoids n = 52), the larvae were injected with ddH_2_O. Parasitism decision procedure and data analysis were as described above.

## Results

### Effect of time interval on development success of superparasitism eggs

**Figure 1:**
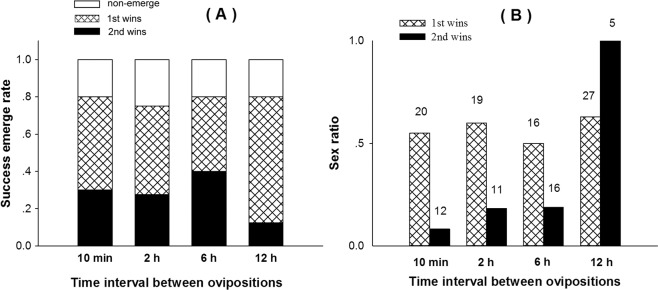
Effect of time intervals between ovipositions on emergence success of the superparasitism eggs. (**A**) The emergence success rate of first and second eggs from 40 oviposition events. (**B**) The sex ratio (proportion of male progeny emerging from contests won by the first and second females) of offspring developed from parasitized (first) eggs or superparasitized (second) eggs. Numbers above bars indicate numbers of individuals in each sample.

Studies using the two different strains of parasitoids showed that eggs of both strains could develop from superparasitism events, but their survival rates varied depending on the time interval between initial oviposition and superparasitism oviposition. There were no significant differences in the rate of successful parasitoid development when parasitoids were exposed at 10 min (χ^2^ = 3.333, df = 1, p = 0.068), 2 h (χ^2^ = 3.413, df = 1, p = 0.065) and 6 h (χ^2^ = 0, df = 1, p = 1) intervals between first and second events (Fig. 1A, Supplementary Figs. 2–5). However, when parasitoid females (FZ strain) attacked *P. xylostella* larvae parasitized by a primary parasitoid (ZJ strain) 12 h before, emergence rate was significantly lower for the superparasitized strain than the primary strain (χ^2^ = 25.208, df = 1, p < 0.001) (Fig. 1A).

The sex ratio of the first egg that developed to adulthood was not influenced by short time intervals of superparasitism. For the interval of 12 h, if the first eggs survives, the sex ratio of parasitized eggs was 0.63 but, if the second eggs survives and develops, it produced only males (superparasitizedeggs), although in very low number (n = 5) (Fig. 1B).

### Effect of superparasitism on progeny production and bionomics of *C. vestalis* offspring

Superparasitism had no adverse effect on progeny production and bionomics in *C. vestalis* (Table 1). Parasitized, self-superparasitized and conspecific-superparasitized offspring showed similar survival rates (Supplementary Table 2). With single parasitism, 62% of emerged individuals were females, and self-superparasitism treatment and conspecific-superparasitism produced 57% and 58%, respectively.

**Table 1:** Life history traits of *C. vestalis* exposed to parasitism, self-superparasitism and conspecific-superparasitism treatments (mean ± SD).

Sex of insect Type of treatment	Number of successfully developed individual^a^	Time to cocoon formation (d)	Time to adult emergence from cocoon (d)	Adult duration (d)	Cocoon weight (mg)	Hind tibia length (mm)	Forewing length (mm)
**Females**							
Parasitism	23	6.1 ± 0.8	4.1 ± 0.7	10.3 ± 5.1	1.78 ± 0.246	0.756 ± 0.050	2.174 ± 0.106
Self-superparasitism	20	6.3 ± 0.7	4.0 ± 0.5	11.4 ± 5.4	1.72 ± 0.202	0.749 ± 0.052	2.150 ± 0.103
Conspecific-superparasitism	21	6.2 ± 0.8	4.1 ± 0.7	9.9 ± 5.1	1.72 ± 0.333	0.755 ± 0.069	2.186 ± 0.110
**Males**							
Parasitism	14	5.8 ± 0.6	4.1 ± 0.9	9.4 ± 5.3	1.72 ± 0.205	0.744 ± 0.034	2.207 ± 0.086
Self-superparasitism	15	6.1 ± 0.6	3.4 ± 0.7	10.7 ± 4.5	1.68 ± 0.190	0.738 ± 0.040	2.152 ± 0.095
Conspecific-superparasitism	15	5.9 ± 0.7	3.8 ± 0.9	9.1 ± 5.1	1.58 ± 0.296	0.715 ± 0.051	2.101 ± 0.143

^a^Individuals that successfully reached adulthood from the initial 60 replicates (including males and females).

### Discrimination between parasitized and unparasitized larvae

#### Comparing rate of oviposition of *C. vestalis* on unparasitized and parasitized *P. xylostella* larvae

When a *C. vestalis* female encountered a host in the glass tube, she became more active, moving very rapidly. This behavior was similar when encountering a unparasitized or parasitized host (Supplementary Videos 2 and 3). She used her antenna to detect the host and decided to accept or reject the host before ovipositing (Supplementary Video 4). On some occasions, the larva could escape when feeling the antennae, suggesting its capacity to detect the parasitoid (Supplementary Video 1).

**Figure 2:**
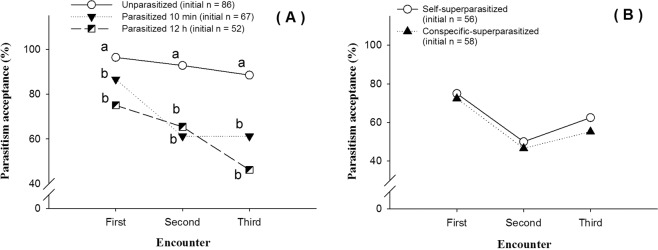
Parasitized host discrimination ability detection of *C. vestalis*. (**A**) Parasitism acceptance rates of *C. vestalis* to unparasitized or parasitized *P. xylostella* larvae, which had been parasitized 10 minutes or parasitized 12 h prior (GLMMs, Treatment main effect: *F* = 34.448, df = 2, P < 0.001; Encounter main effect: *F* = 11.658, df = 2, *P* < 0.001; Treatment × Encounter: *F* = 0.838, df = 4, *P* = 0.501); Different letter in same encounter indicate significant difference (*P* < 0.017), Bonferroni test. (**B**) Parasitism acceptance rates of *C. vestalis* to self-parasitized and conspecific-parasitized *P. xylostella* larvae (GLMMs, Treatment main effect: *F* = 0.035, df = 1, *P* = 0.851, Encounter main effect: *F* = 8.925, df = 2, *P* < 0.001; Treatment × Encounter: *F* = 0.039, df = 2, *P* = 0.961).

Acceptance rate was the highest at the first encounter event when exposed to three larvae sequentially in all treatments (Fig. 2A). The acceptance rate, however, declined significantly over time with the third exposure having the lowest value (Fig. 2A). Acceptance rates were significantly different between treatments (P < 0.001), and when the parasitoids were exposed to unparasitized larvae, they exhibited the highest acceptance rates.

#### Discrimination of *C. vestalis* female between self-parasitized and conspecific-parasitized *P. xylostella* larvae

Female *C. vestalis* did not discriminate between larvae that were self-parasitized and conspecific-parasitized (Fig. 2B).

### Effect of topical hemolymph application on parasitism acceptance or rejection rate of inexperienced parasitoid females

**Figure 3:**
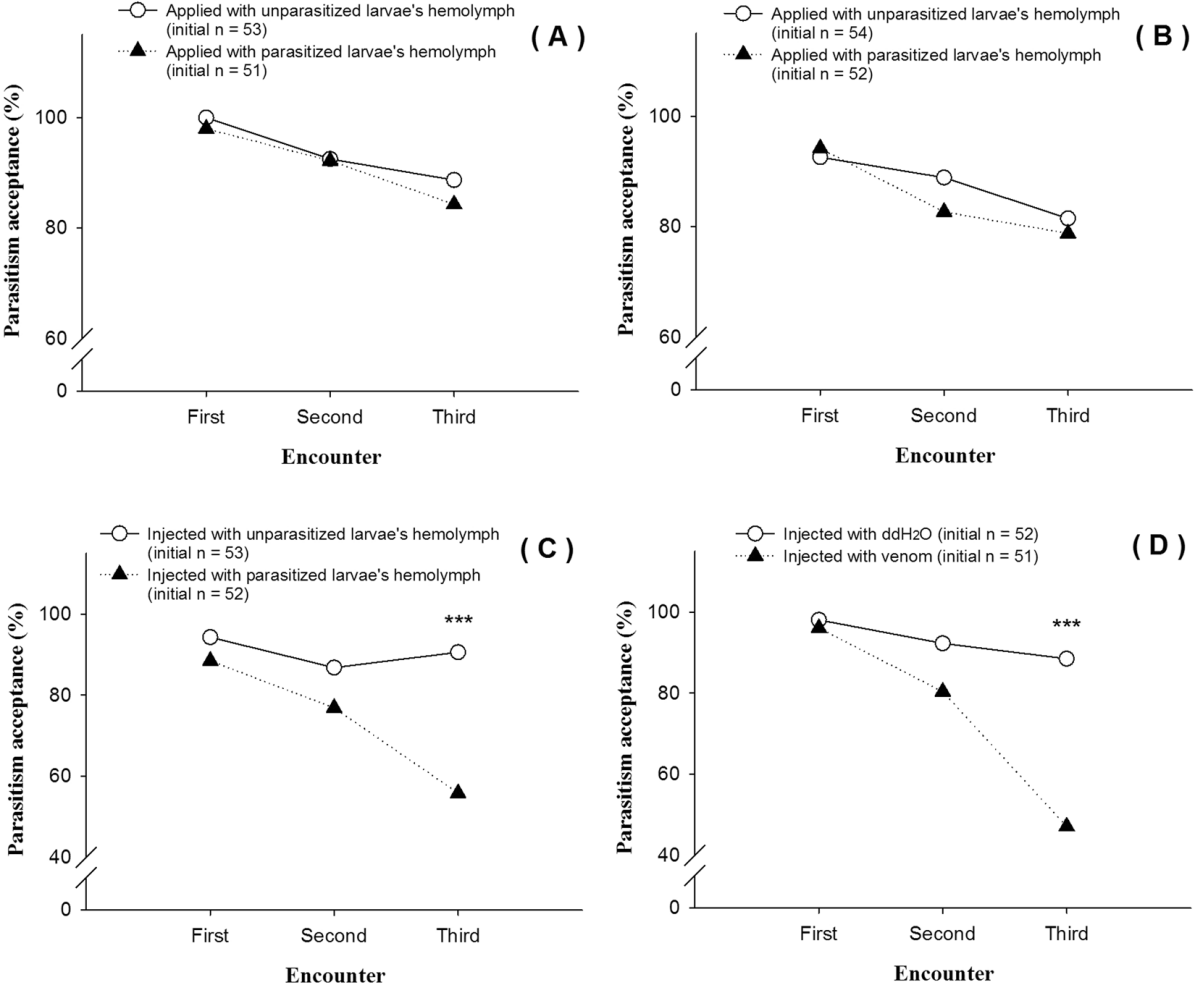
Results of the host discrimination experiments (sections 1.4, 1.5, 1.6 and 1.7). (**A**) Parasitism acceptance rates of inexperienced *C. vestalis* to unparasitized *P. xylostella* larvae with odor of parasitized larvae’s hemolymph (GLMMs, Treatment main effect: *F* = 0.258, df = 1, *P* = 0.612; Encounter main effect: *F* = 3.612, df = 2, *P* = 0.028; Treatment × Encounter: *F* = 0.095, df = 2, *P* = 0.909). (**B**) Parasitism acceptance rates of experienced *C. vestalis* to unparasitized *P. xylostella* larvae with odor of parasitized larvae’s hemolymph (GLMMs, Treatment main effect: *F* = 0.172, df = 1, *P* = 0.678; Encounter main effect: *F* = 4.117, df = 2, *P* = 0.017; Treatment × Encounter: *F* = 0.348, df = 2, *P* = 0.706). (**C**) Parasitism acceptance rates of inexperienced *C. vestalis* to unparasitized *P. xylostella* larvae which injected with parasitized larvae’s hemolymph (GLMMs, Treatment main effect: *F* = 8.898, df = 1, *P* = 0.003, Encounter main effect: *F* = 3.508, df = 2, *P* = 0.031; Treatment × Encounter: *F* = 1.902, df = 2, *P* = 0.151); The Chi-square test for each encounter: First encounter: χ^2^ = 1.157, df = 1, *P* = 0.282; Second encounter: χ^2^ = 1.725, df = 1, *P* = 0.189; Third encounter: χ^2^ = 16.252, df = 1, *P* < 0.001. (**D**) Parasitism acceptance rates of inexperienced *C. vestalis* to unparasitized *P. xylostella* larvae which injected with parasitoid’s venom solution (GLMMs, Treatment main effect: *F* = 6.378, df = 1, *P* = 0.012, Encounter main effect: *F* = 8.937, df = 2, *P* < 0.001; Treatment × Encounter: *F* = 1.252, df = 2, *P* = 0.287); The Chi-square test for each encounter: First encounter: χ^2^ = 0.364, df = 1, *P* = 0.546; Second encounter: χ^2^ = 3.113, df = 1, *P* = 0.078; Third encounter: χ^2^ = 20.270, df = 1, *P* < 0.001.

Hemolymph volatiles did not affect parasitism acceptance rate of *C. vestalis*. Females showed similar acceptance rates when exposed to unparasitized larvae topically treated with hemolymph of parasitized of *P. xylostella* larvae (*P* = 0.612). Of the initial 53 inexperienced parasitoids exposed to larvae with unparasitized hemolymph, 53 were accepted in the first minute, 49 during the second minute and, 47 in the third minute. Numbers were similar when exposed to parasitized larvae (initial parasitoids = 51, first minute, 50 accepted, second minute, 47 and third minutes 43) (Fig. 3A).

### Effect of topical hemolymph application on parasitism acceptance or rejection rate of experienced parasitoid females

Like in the case of inexperienced parasitoids, experienced females showed similar responses to unparasitized larvae applied with hemolymph of parasitized of *P. xylostella* larvae (*P* = 0.678). Hemolymph volatiles did not affect parasitism acceptance rate of experienced *C. vestalis* (Fig. 3B).

### Influence of injected hemolymph extract from parasitized larvae on parasitism acceptance or rejection rate of inexperienced parasitoids

The injection of hemolymph from parasitized larvae or unparasitized larvae into unparasitized larvae significantly influenced inexperienced parasitoid host acceptance. Larvae injected with the hemolymph extract from unparasitized larvae had a significantly higher acceptance rate by *C. vestalis* females (n = 53) than those treated with hemolymph from parasitized hosts (*P* = 0.003) (Fig. 3C). While the rate of parasitism acceptance remained relatively constant for the three minutes for unparasitized larvae, there was a significant decline over time for the larvae injected with parasitized larva’s hemolymph.

### Effect of parasitoid venom on parasitism acceptance/rejection rate of inexperienced parasitoids

When a parasitoid encountered the first larva, the acceptance rate did not significantly differ whether the larva was injected with parasitoid venom or water (Fig. 3D). While acceptance rates remained relatively constant over time for larvae injected with water, parasitoids sequentially exposed to larvae injected with venom showed a decline in acceptance rate over the three minutes (Fig. 3D).

## Discussion

In this study, we showed that superparasitism did not necessarily have adverse effect on the parasitoid. Superparasitized hosts could support parasitoid development to adulthood at a similar rate to that of unparasitized hosts. Importantly, however, this was the case only when superparasitism occurred 6 h or less after the initial parasitism event. At 12 h there was a marked cost of superparasitism in terms of reduced emergence and male dominated progeny. Parasitoid foraging behavior and egg production strategies greatly vary among parasitoid species^38,39^. In solitary parasitoids, superparasitism may be disadvantageous when larvae from eggs laid later must compete with the older conspecifics, and then are less likely to develop to adulthood^40^. Superparasitism may only be considered adaptive if the second eggs maintain similar rates of survival and growth than the first eggs^41^ and as shown in our study.

Microsatellites as molecular markers are increasingly used to study genetic diversity in natural pest enemy populations^42^. Based on seven microsatellites, we used two laboratory *C. vestalis* strains (ZJ-strain and FZ-strain) coming from different populations to determine the success rates of first and second eggs to emerge and found that eggs laid by FZ parasitoids could successfully develop to adulthood at a similar emerge rate to primary eggs (laid by ZJ strain) for the first three time intervals (10 min, 2 and 6 h). Superparasitism may have adverse effects on biological traits of parasitoids. For instance, in *Anaphes nitens*, it leads to more male offspring^43^. In the present study, the sex ratio of the first egg was not influenced by superparasitism, but the sex ratio of the superparasitized egg was obviously changed depending on the time interval between oviposition events. We assume that, when the time interval between first and second oviposition extends to 12 h, few second eggs survive over the first egg. Moreover, due to males developing faster than females, when superparasitism occurs (interval time >12 h), female eggs from second oviposition may have no chance to survive.

Superparasitism may also cause other adverse effects on biological traits of parasitoids. For example, it can cause smaller body size and longer development time in *Cotesia flavipes*^5^. High level of superparasitism (superparasitism more than twice) in *C. vestalis* can lead to longer immature duration and more male offspring^29^. However, when low level of superparasitism was investigated, we did not detect such adverse effects in life history traits of the progeny including immature duration, adult duration and body size. We hypothesized that the few adverse effects in low level of superparasitism might be an adaptive trait of *C. vestalis* and suggest low level superparasitism in rearing this parasitoid could be acceptable.

Superparasitism was clearly not advantageous for *C. vestalis* when the time interval between oviposition events was 12 h. The emergence success of the second egg was reduced after 12 h and likely due to the first egg having enough time to develop to a sufficiently advanced stage such that this individual was able to compete successfully against the second-laid individual. Our finding is consistent with a study by Zhang *et al*.^44^ that report, through dissection of the superparasitised *P. xylostella* larvae, rates of first egg success of 86.7% after 24 h and 95.0% after 48 h^44^. In general, the probability of a second egg competing and emerging depends on time elapsed since the first parasitism in solitary parasitoids^45^. This similar phenomenon has been observed in other gregarious and solitary parasitoid species, such as *Bracon hebetor*^46^, *Metaphycus flavus*^47^, *Echthrodelphax fairchildii*^48^, and *Trissolcus basalis*^49^.

In the present study, the highest rate of host acceptance was during the first encounter between a parasitoid and a potential host. Inexperienced *C. vestalis* were highly likely to parasitize the first encountered host whether already parasitized or not. This phenomenon may be adaptive, because the naïve parasitoid’s encounter with the initial host potentially represents the only host, she will ever encounter given that hosts may be scare, and she may die before encountering another one. Encounters with subsequent hosts signal the likelihood that she will find others before death so can afford to be progressively more selective and less accepting of previously parasitized host. Host selection is an important factor for parasitoid survival^1^. The quality and availability of the host (i.e. resources for egg development) is a key issue for the fitness of parasitoids^9^. In the solitary parasitoid *Diachasmimorpha longicaudata* (Hymenoptera: Braconidae), for example, when the host density fluctuates between high and low per day, high superparasitism rate is associated with the low host density^50^. In *Cotesia flavipes*, the naïve females treat the parasitized host similar to unparasitized host, but the experienced females reject the parasitized host^5^.

Our results showed that when *C. vestalis* was provided successively with more hosts, acceptance rates of parasitized hosts declined. Thus, *C. vestalis* showed some capacity for discrimination between parasitized and unparasitized hosts, as it has been seen for other species^51–54^. For solitary parasitoids, previously parasitized hosts are referred to as low quality, and foraging theory predicts that the parasitoid should reject superparasitism^4^.

Our results strongly suggest that *C. vestalis* can discriminate parasitized *P. xylostella* from unparasitized one and studies with venom and hemolymph treated hosts illustrate the mechanism of host discrimination. Various parasitoids can use pheromones for host discrimination, such as heptanone and alkadienes to avoid already parasitized larvae^14,21,55^ and these are detected by the parasitoid antennae^17^. Our videos showed *C. vestalis* using their antennae on host larvae, there was no discrimination between self-parasitized and conspecific-parasitized hosts, which contrasts with *Muscidifurax zaraptor*^40^, *Echthrodelphax fairchildii*^56^, and *Aphelinus asychis*^57^. We propose that *C. vestalis* use internal cues for parasitized host discrimination, because injecting parasitoid host hemolymph or parasitoid venom into unparasitized hosts altered *C. vestalis* acceptance. This did not occur in response to topically applied hemolymph. This contrasts with other species such as *Anaphes victus* (Hymenoptera: Mymaridae) that can determine host status based on antennal contact^22^, and similar to *Dolichogenidea tasmanica* (Hymenoptera: Braconidae), which discriminated host status by ovipositor injection^58^.

Irrespective of the potential for *C. vestalis* to determine the parasitism status of a host larvae, this may be overridden by the need to make a very rapid decision to oviposit. *P. xylostella* larvae were able to escape from parasitoids during some encounters even in the enclosed conditions of the present tests. In the field, larvae have the additional option of dropping from the plant to escape danger^59^. This risk to the parasitoid of losing contact with vigorous host larvae that have effective behavioral defenses may have favored selection for rapid acceptance of hosts immediately after encounter, albeit with the tradeoff of a fitness cost should that host already be parasitized.

In conclusion, *C. vestalis* exhibits limited capacity for avoidance of superparasitism, even though it can discriminate between unparasitized and already parasitized hosts, because discrimination requires the insertion of the ovipositor into a vigorously mobile host that is likely to escape unless rapidly utilised. Recognition that a host is already parasitized seems to require first the immediate insertion of the ovipositor and the female parasitoid needs to decide very rapidly whether to deposit an egg. As a new experienced female, she will use her sensory apparatus to apply some level of host discrimination in subsequently-encountered hosts. The cost of this limited discrimination capacity appears to be low if the time interval between parasitism events is low (minutes) but, under longer and biologically realistic time intervals (hours, and presumably days) there is a clear cost of superparasitism. The extent to which this behavior may be adaptive or not for parasitoids under field conditions where hosts have the chance of escaping if not rapidly parasitized, remains to be investigated.

## Supplementary information

Supplementary files.

Supplementary video1.

Supplementary video2.

Supplementary video3.

Supplementary video4.
