# Supplementary material for: Mechanism and consequences for avoidance of superparasitism in the solitary parasitoid Cotesia vestalis
Source: Sci Rep. 2020 Jul 10;10:11463. doi: 10.1038/s41598-020-67050-1 (PMC7351760; doi:10.1038/s41598-020-67050-1)
Supplement: Supplementary file 1 — Supplementary files. [file 41598_2020_67050_MOESM1_ESM.pdf]

SUPPLEMENTARY MATERIAL

Mechanism and consequences for avoidance of superparasitism in the solitary parasitoid *Cotesia vestalis*

Wen-bin Chen[1,2], Liette Vasseur[1,3], Shuai-qi Zhang[1,2], Han-fang Zhang[1,2], Jun Mao[1,2], Tian-sheng Liu[1,2], Xian-yong Zhou[1,2], Xin Wang[1,2], Jing Zhang[1,2], Min-sheng You[1,2], Geoff M. Gurr[1,4]

[1]State Key Laboratory of Ecological Pest Control for Fujian and Taiwan Crops, Institute of Applied Ecology, Fujian Agriculture and Forestry University, Fuzhou, China, [2]Joint International Research Laboratory of Ecological Pest Control, Ministry of Education, Fuzhou 350002, China, [3]Department of Biological Sciences, Brock University, St. Catharines, Ontario, Canada, [4]Graham Centre, Charles Sturt University, Orange, New South Wales, Australia

Offspring strain analysis of four interval superparasitism treatments by microsatellites.

Total DNA of *Cotesia vestalis* was individually isolated using the DNeasy Blood and Tissue Kit (QIAGEN, Hilden, Germany) following the manufacturer's instructions.

Seven microsatellites developed by us were chosen for this study and primer sequences are shown in Supplementary Table 1. For every microsatellite locus, the PCR reaction was performed in a 25 μL mixture, containing 12.5 μL Mix (Promega), 0.2 μL forward primer, 0.8 μL reverse primer, and 0.8 μL *M-13*. The temperature was set at 94°C for 10 min, and then 30 cycles at 94°C for 30 s, Tm (the optimal annealing temperatures, see Table 1) for 45 s, 72°C for 45 s, followed by 8 cycles at 94°C for 30 s, 53°C for 45 s, 72°C for 45 s, and finally at 72°C for 10 min. The amplification products were detected using an ABI 3730 sequencer (Applied Biosystems) and analyzed using the GeneMapper 4.1 (Applied Biosystems).

Cluster analysis was first used to evaluate whether the seven microsatellites data could separate maternal strains of ZJ and FZ. Cluster analysis was performed using the method of principal coordinate analysis (PCoA), which is based on the standardized genetic distance matrix between populations. Secondly, the microsatellite data of offspring were individually added into the cluster analysis system. Based on PCoA, we assessed whether the offspring individual belonged to ZJ or FZ strain by visualizing population differentiation. This process was performed using the GENALEX v6.5.

The cluster analysis showed that: in the 10 min treatment (offspring represented by 20 females and 12 males), 11 females and 1 male corresponded to FZ maternal strain, meanwhile the remaining 20 belonged to the progeny of ZJ maternal strain; in the 2h treatment (offspring had 16 females and 14 males), 9 females and 2 males were the progeny of FZ maternal strain; in 6h treatment (offspring represented by 21 females and 11 males), 13 females and 3 males were the progeny of FZ maternal strain; and in the

12h treatment (offspring had 10 females and 22 males), only 5 males were descendants of the FZ maternal strain.

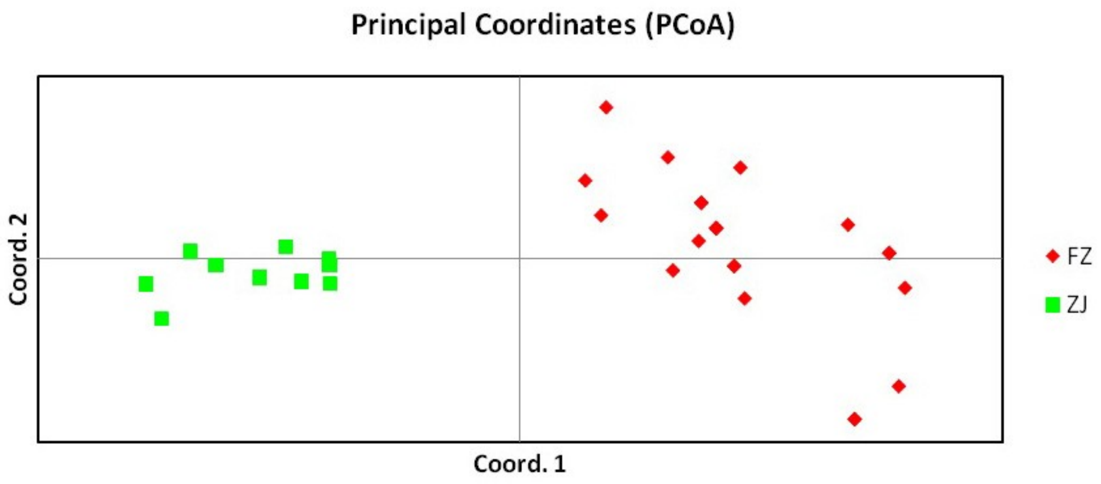

Supplementary Figure 1. The PCoA analysis based on standardized genetic distance data across 7 polymorphic SSR loci successfully separated between ZJ and FZ parasitoid strains. The first two coordinates explained 55.4% and 12.0% of the total variance.

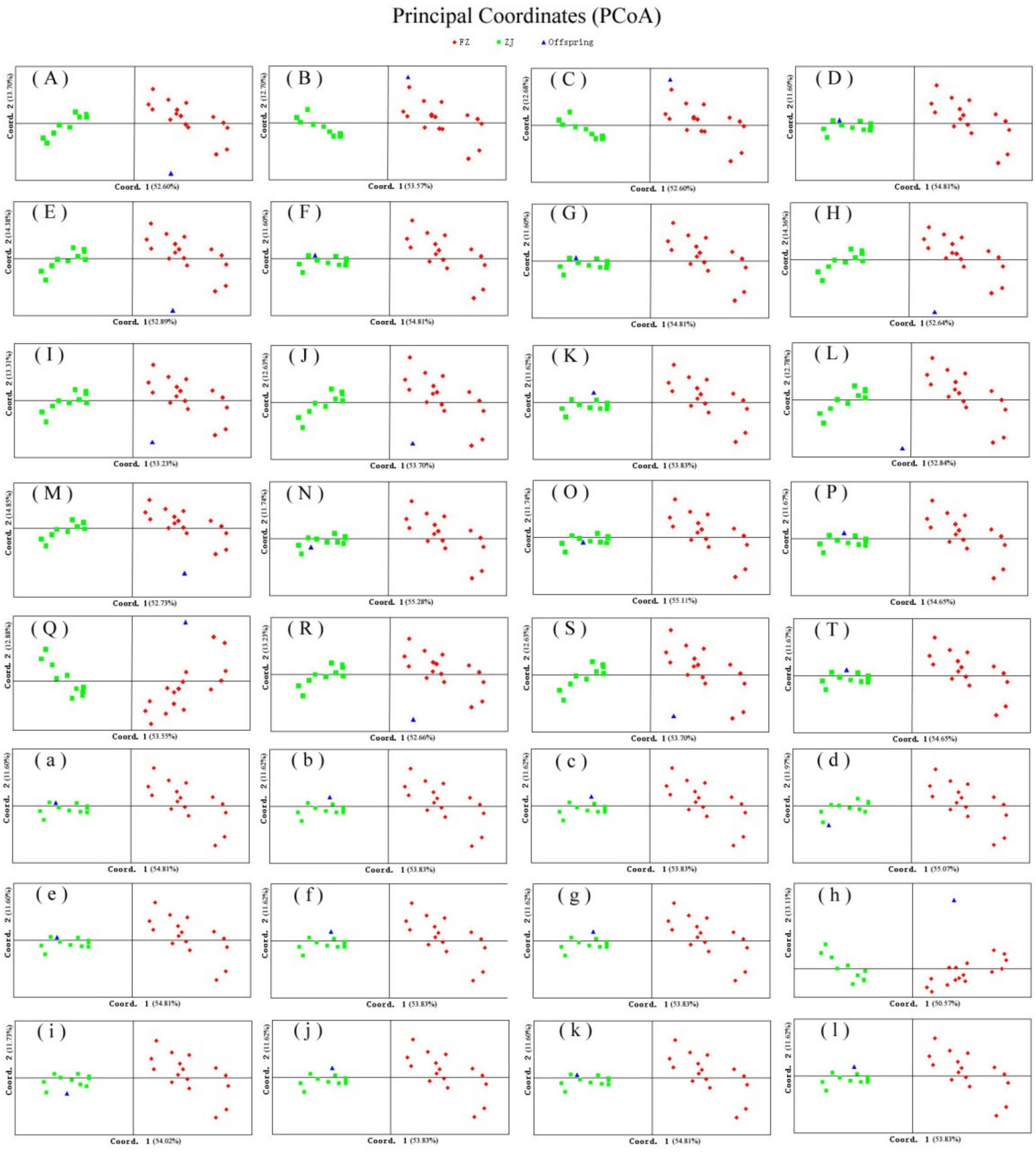

Supplementary Figure 2. The PCoA analysis based on standardized genetic distance matrix between ZJ and FZ maternal strains and individual offspring from the 10 min treatment. (A-T: female offspring, a-l: male offspring)

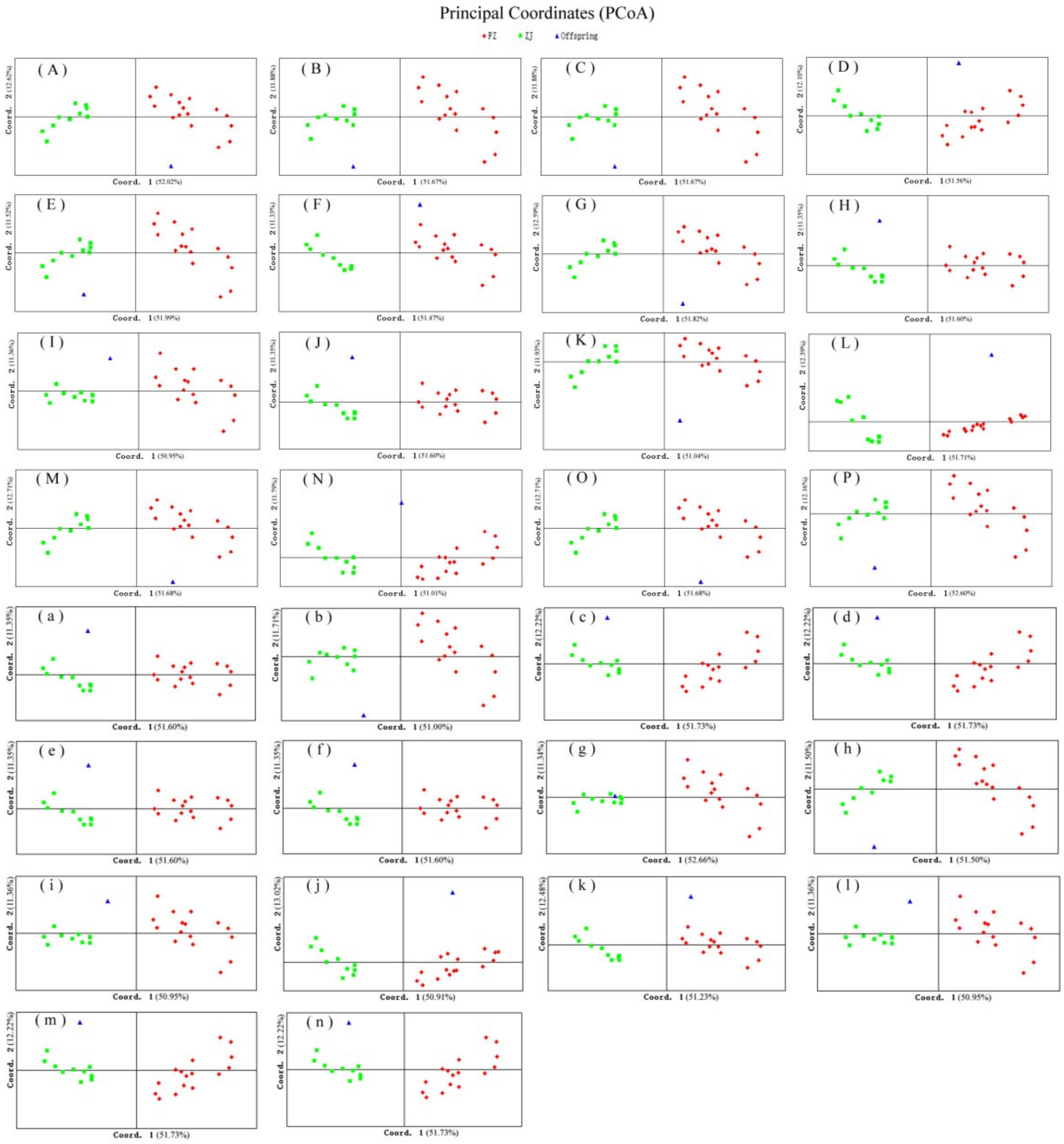

Supplementary Figure 3. The PCoA analysis based on standardized genetic distance matrix between ZJ and FZ maternal strains and individual offspring from the 2h treatment. (A-P: female offspring, a-n: male offspring)

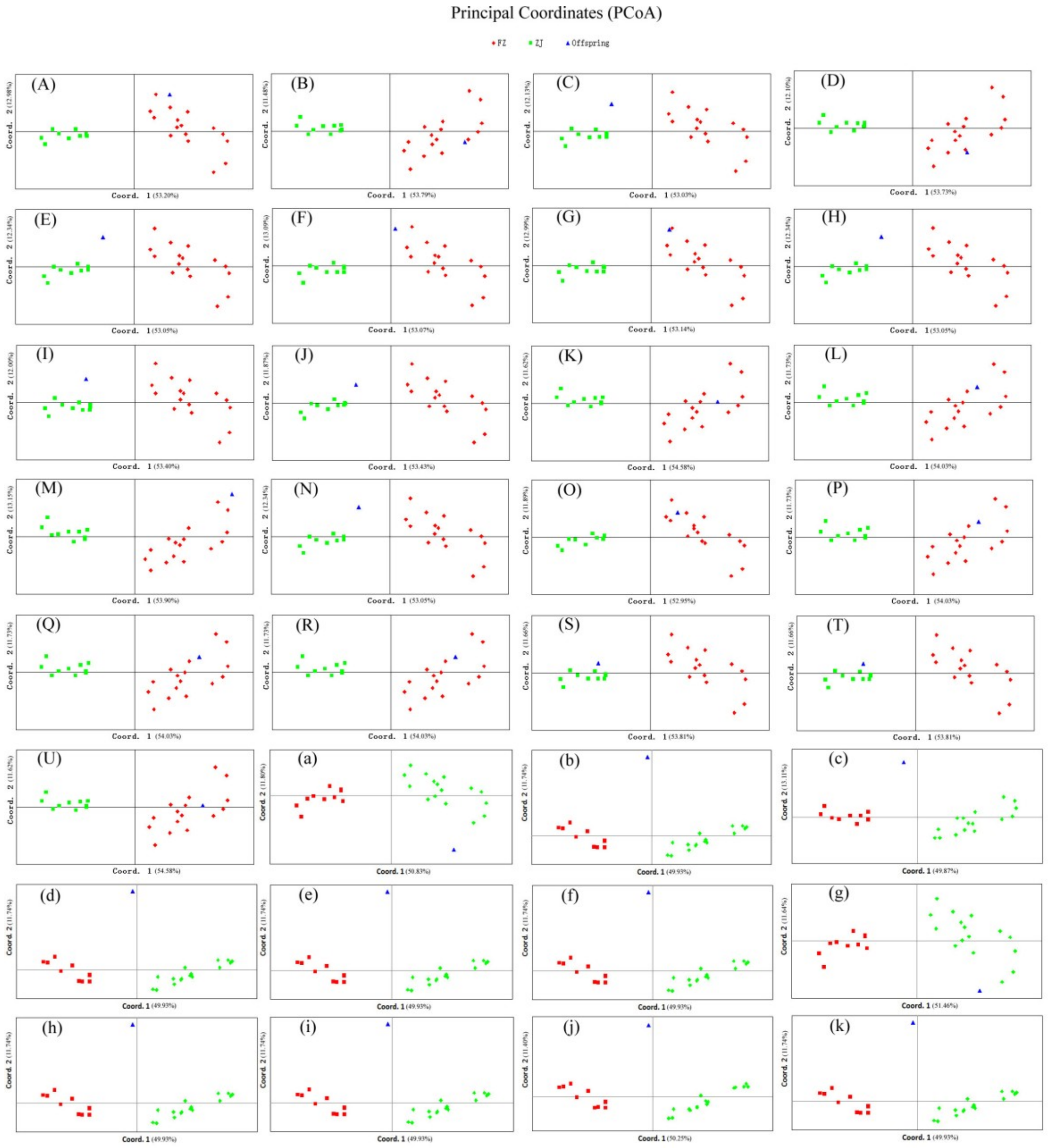

Supplementary Figure 4. The PCoA analysis based on standardized genetic distance matrix between ZJ and FZ maternal strains and individual offspring from the 6h treatment. (A-U: female offspring, a-k: male offspring)

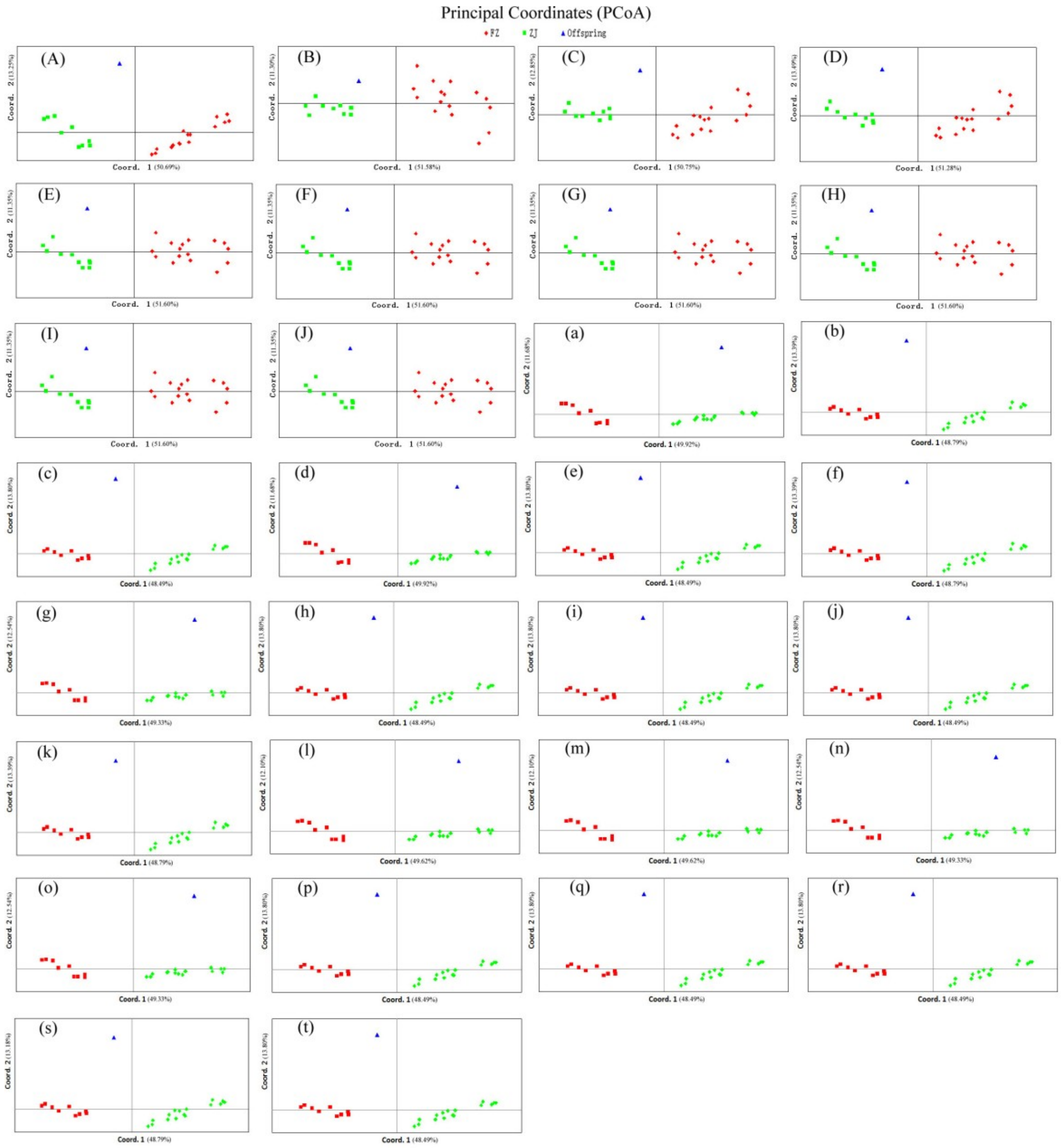

Supplementary Figure 5. The PCoA analysis based on standardized genetic distance matrix between ZJ and FZ maternal strains and individual offspring from the 12h treatment. (A-J: female offspring, a-t: male offspring)

Supplementary Table 1. Charaterization of the 7 polymorphic microsatellite loci developed for *C. vestalis*.

Locus	Repeat motif	Primer sequences（5'-3'）	*Tm*(℃)	*Na*	Size range(bp)	H_O/H_E	Fis	Annotation
C6	$(CTG)_6$	F：AGAGCGGCAGTATCGTGAGT<br>R：AGGAAAAGTCCTCAGCCTCC	53	5	245-260	0.163/0.312	0.4807*	Putative uncharacterized protein
C19	$(AAT)_5$	F：CGCGAAAGAACGAATTTGAG<br>R：TCACAGTATACGTCATTCCCAAG	54	5	129-141	0.092/0.156	0.4138*	Bifunctional protein FolD
C21	$(AAT)_5$	F：TCGCTAGAAAAAGTTTCGGC<br>R：AATGAAGCAGGGTGAAATGC	53	4	232-241	0.112/0.137	0.1852	Putative uncharacterized protein
C22	$(CTG)_6$	F：CGCGACTCTCTGGCTCTACT<br>R：TCAGGAGTCAGGAGTGGCTT	56	3	155-161	0.143/0.242	0.4121*	cAMP responsive element-binding protein-like 2
C31	$(GAA)_6$	F：AAAACGTGACCAAAAGCTGG<br>R：GGCCCGAGTACAAACAACTC	55	2	215-218	0.092/0.116	0.2117	Putative uncharacterized protein
C32	$(CTG)_6$	F：TATGGGCGATAAAGGTGCTC<br>R：AGGAAAAGTCCTCAGCCTCC	55	4	291-300	0.153/0.279	0.4541*	Ceramide kinase
C57	$(TCG)_6$	F：CCGGAACTGTTTTGTCACG<br>R：CCGGAGTACGCTCTCAAGAC	52	4	124-133	0.173/0.256	0.3245*	Putative uncharacterized protein

Tm annealing temperature of primer pairs, *Na* number of alleles, *Ho* observed heterozygosity, H_E expected heterozygosity

* Significant deviation from Hardy–Weinberg equilibrium ($P < 0.05$)

Supplementary Table 2. Survival rate of *C. vestalis* progeny from parasitism, self-superparasitism and conspecific-superparasitism.

Treatment	Survival rate (%)	
	Egg and juvenile	cocoon
Parasitism (n=60)	75.0	82.2
Self-superparasitism (n=60)	66.7	87.5
Conspecific-superparasitism (n=60)	76.7	78.3
